# Electron momentum densities near Dirac cones: Anisotropic Umklapp scattering and momentum broadening

**DOI:** 10.1038/s41598-017-00628-4

**Published:** 2017-04-03

**Authors:** N. Hiraoka, T. Nomura

**Affiliations:** 1 National Synchrotron Radiation Research Center (NSRRC), 101 Hsin-Ann Road, Hsinchu, 30076 Taiwan; 2 National Institutes for Quantum and Radiological Science and Technology (QST), SPring-8, 1-1-1 Kouto, Sayo, Hyogo 679-5148 Japan

## Abstract

The relationship between electron momentum densities (EMDs) and a band gap is clarified in momentum space. The interference between wavefunctions *via* reciprocal lattice vectors, making a band gap in momentum space, causes the scattering of electrons from the first Brillouin zone to the other zones, so-called *Umklapp* scattering. This leads to the broadening of EMDs. A sharp drop of the EMD in the limit of a zero gap becomes broadened as the gap opens. The broadening is given by a simple quantity, *E*_*g*_/*v*_*F*_, where *E*_*g*_ is the gap magnitude and *v*_*F*_ the Fermi velocity. As the ideal case to see such an effect, we investigate the EMDs in graphene and graphite. They are basically semimetals, and their EMDs have a hexagonal shape enclosed in the first Brillouin zone. Since the gap is zero at Dirac points, a sharp drop exists at the corners (K/K’ points) while the broadening becomes significant away from K/K’s, showing the smoothest fall at the centers of the edges (M’s). In fact, this unique topology mimics a general variation of the EMDs across the metal-insulator transition in condensed matters. Such an anisotropic broadening effect is indeed observed by momentum-density-based experiments *e*.*g*. x-ray Compton scattering.

## Introduction

The relationship between electron momentum densities (EMDs) and a band gap has been little discussed in reciprocal space or momentum space, despite a fundamental importance in condensed matter physics. As is well known, a fundamental characteristic of electrons in metals is to form the Fermi surface which defines the boundary between occupied and unoccupied states in momentum space^1^. In contrast to this, in semiconductors or insulators, the highest occupied states coincides with Brillouin zone boundaries, on which the energy gap opens due to the interference between the wavefunctions in the first Brillouin zone and higher zones, leading to the disappearance of the Fermi surface. If the electron band or the dispersion specified by momentum and energy *ψ*(**p**, *E*) is projected onto the *E* axis, we obtain the density of states (DOS) *ρ*(*E*); On the other hand, if *ψ* is projected onto **p** space, we have an electron momentum density (EMD) *ρ*(**p**) ≡ *ρ*(*p*_*x*_, *p*_*y*_, *p*_*z*_). Consequently, there emerges an energy gap in the DOS when the system transits from a metal to an insulator. However, it has rarely been discussed what happens to the EMD: One may anticipate that Fermi-surface-associated features could remain even after the transition to an insulator if the band gap is narrow, while it is unlikely in wide-gap materials. It is prerequisite to know how EMD evolves during such a transition upon performing experimental and theoretical studies based on momentum space. The problem has implicitly been suggested so far^2–5^. Nonetheless, crucial evidence, particularly in experiments, is still lacking and thus the issue is far from a general consensus. In fact, the electronic structures in graphene and graphite ideally possess both aspects of a metal and an insulator. The electrons behave as in a metal at the Dirac points (*K*/*K*′ points) in momentum space, while behaving as in an insulator away from those points. Because of this, the EMD has a unique topology in those materials. Here we show that the characteristic behaviors are indeed observable by an x-ray Compton scattering experiment with an enhanced momentum resolution.

**Figure 1:**
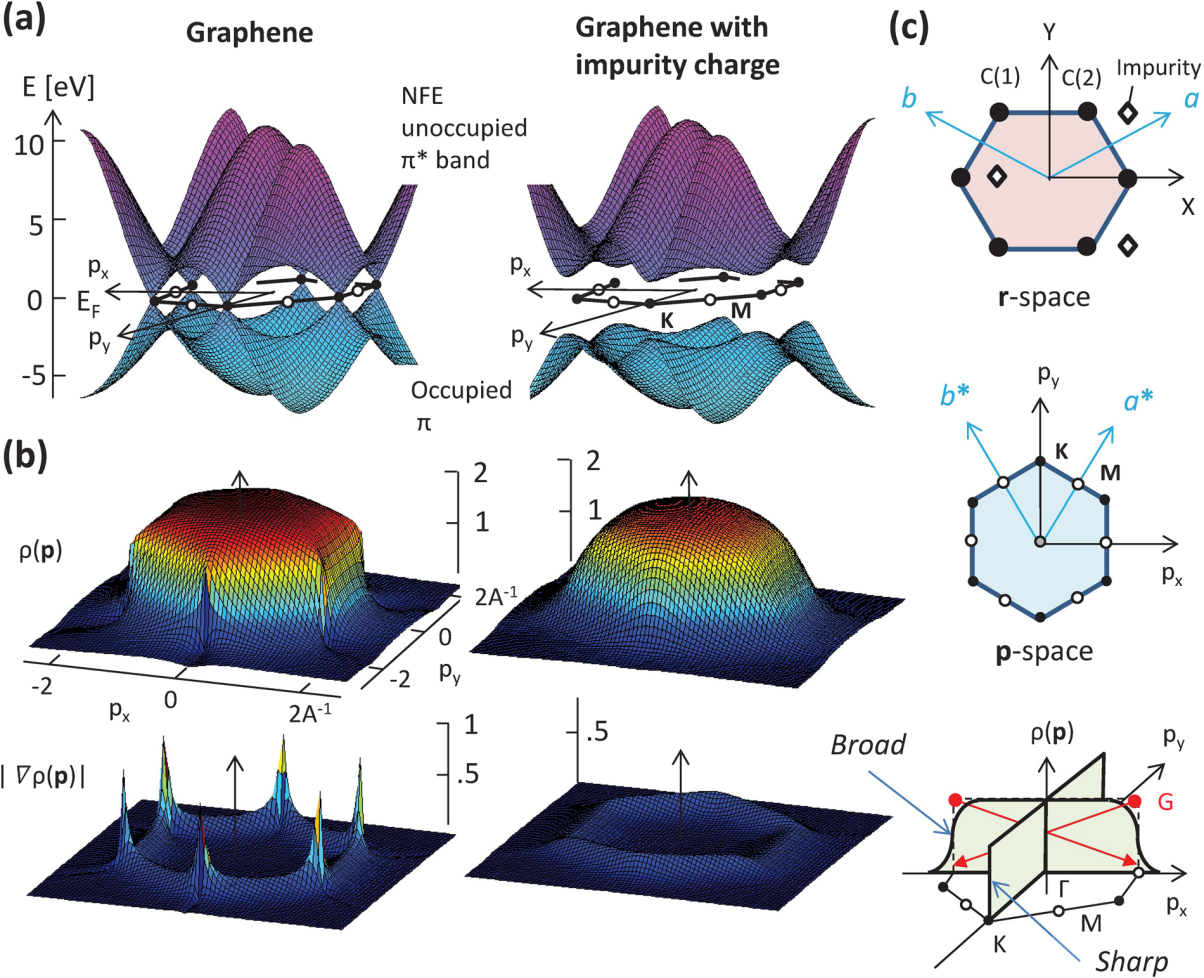
(**a**) Occupied *π* and unoccupied π* bands computed for the nearly-free-electron model, both without (left) and including impurity charges (right): Impurity charges break the symmetry, resulting in a wide energy gap over momentum space. (**b**) Electron momentum densities (EMDs) and the derivatives in graphene show an anisotropic broadening (left) while those with impurity charges exhibit a nearly isotropic broadening (right). (**c**) Positions of charges [original (•) and impurity (◊)] in real space (top), First Brillouin zone (middle), Anisotropic Umklapp scattering (bottom).

Among carbon-based materials, graphene has the simplest form, but exhibits the most fascinating properties^6^. Graphene is a monolayer having a honeycomb structure, and thus is completely two-dimensional (2D). Two linear bands cross without interference at the Dirac points (*K*/*K*′), where three Brillouin zones share the corners, forming the so-called Dirac cone (see Fig. 1a). Owing to this linearity, there emerge massless fermions, displaying various anomalies, such as ultra-thin ballistic transport properties^7^, the half-integer quantum Hall effect^8,9^, and so on^10–13^. Whereas, at the *M* points, on which neighboring zones share the edges, the valence and the conduction bands are well separated, exhibiting an energy gap as large as 4 eV. One therefore finds an EMD variation as a function of a band-gap magnitude along the *K*(*K*′)-*M* axis. The electronic transport only occurs near the Dirac cones while there is no contribution from elsewhere in the Brillouin zone. Distinctive electronic properties would be observed at *K*.

## Results

The behavior of the EMD can be examined in a quantitative way using the nearly-free-electron (NFE) model^14–16^. The model is simple but sufficient for the present purpose, and could even be considered ideal because other effects (such as contributions from wavefunctions in muffin-tin potentials or fictitious features introduced during the transformation from the reduced zone scheme to the extended zone scheme) are completely excluded, making the origin of the effect we wish to describe unambiguous. Two electrons are introduced as *π* electrons into the potential formed by a periodic arrangement of atoms with a charge *Ze*, where *e* is the elementary charge while *Z* is a magnification parameter, currently set to be 1.0. Their wave functions are expressed by linear combinations of plane waves. The NFE model reproduces the electronic structure in graphene remarkably well. Figure 1a displays the *π* and π* band dispersions, in which the Dirac cones are clearly seen at the *K* points (left-hand side). Figure 1b indicates the EMD *ρ*(**p**) and the derivatives $|\nabla \rho ({\bf{p}})|$. It is seen that the hexagonal EMD enclosed in the first zone has a sharp drop, so-called *Fermi break* ^17–19^ at *K*, in contrast to a smooth fall at *M*. This is even more clearly recognized as a diverging behavior in the $|\nabla \rho ({\bf{p}})|$ map. The unique topology appears prominently if it is compared with the case of a wide-gap insulator, which is obtained by adding impurity atoms, *e*.*g*. of *Z* = 0.5, to lower the crystalline symmetry (Fig. 1a,b, right hand side). Figure 1c illustrates the process of the broadening: Along the Γ-*M* (*p*_*x*_) direction, a portion of EMD leaks from the first zone to the second or higher zones by the scattering due to reciprocal lattice vectors, the so-called *Umklapp* scattering. Whereas, there is no such scattering along the Γ-*K* (*p*_*y*_) direction, and the EMD entirely remains in the first zone.

**Figure 2:**
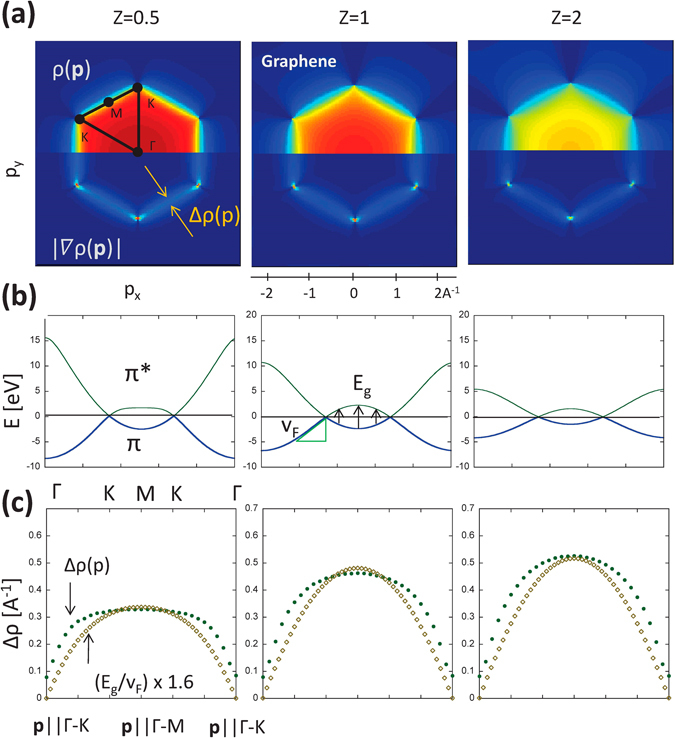
(**a**) EMDs and the derivatives as a function of the charge strength *Z*, (**b**) band dispersions, and (**c**) comparisons between the momentum broadening Δ*p* (FWHM in $\partial \rho (p)/\partial p$ along the radial direction) and the gap magnitude (*E*_*g*_).

Figure 2 shows EMDs as a function of the strength of the periodic potential, *Ze*. The EMDs are computed for *Z* = 0.5 and 2, in addition to *Z* = 1, which well reproduces the tight-binding or first-principles band-structures in literatures^6,20^. The EMDs (**a**), the band dispersions (**b**), and the EMD broadening compared with the gap magnitude (**c**) are displayed. Effectively, we find a relationship of ${\rm{\Delta }}p\sim \eta ({E}_{g}/{v}^{\ast })$, where *η* is a scaling constant. v* is defined as an effective Fermi velocity, *i*.*e*., ${v}^{\ast }={\partial E/\partial p|}_{p\to ZB}$, a gradient of a parabolic dispersion extrapolated to the zone boundary (ZB). The electron dispersion is given by $E={p}^{2}/\mathrm{(2}{m}^{\ast })$ around the zone center but the effective mass m* is not constant and thus v* is difficult to determine in an exact way. We use *v*_*F*_ at *K* for v*. We find that $\eta ({E}_{g}/{v}^{\ast })$ fits to Δ*p* fairly well when *η* = 1.6, corresponding to the full-width-at-half-maximum (FWHM) of the Gaussian function, exp(−*x* ^2^), where *x* = *E*_*g*_/(v*Δ*p*). In reality, *η* varies between 1 and 2. (The non-zero Δ*p* at *K* is due to the discretization in the computation). Such uncertainty can arise from the difficulty in evaluating v* in an exact way since it is not constant.

Having clarified the EMD in graphene, it is straightforward to deduce that in graphite. The latter consists of ABA stacked graphene sheets. Just like bilayer graphene, graphite shares many of properties of graphene but has the following differences^21,22^. First, graphene has the bands linearly crossing at *K*, leading to m* → 0 with finite *v*_*F*_. Graphite has similar bands but they no longer linearly cross and make a van-Hove singularity, leading to *v*_*F*_ → 0 with finite m*^10,11,23^. Second, there are other parallel *π* and π* bands due to lifted degeneracy, exhibiting an energy gap of ~1 eV^20,22^. Nonetheless these differences would only make minor modifications, such as an additional small broadening. The major topology of the EMD would be common between graphite and graphene, appearing like a hexagon having the anisotropic broadening as seen in the NFE model.

**Figure 3:**
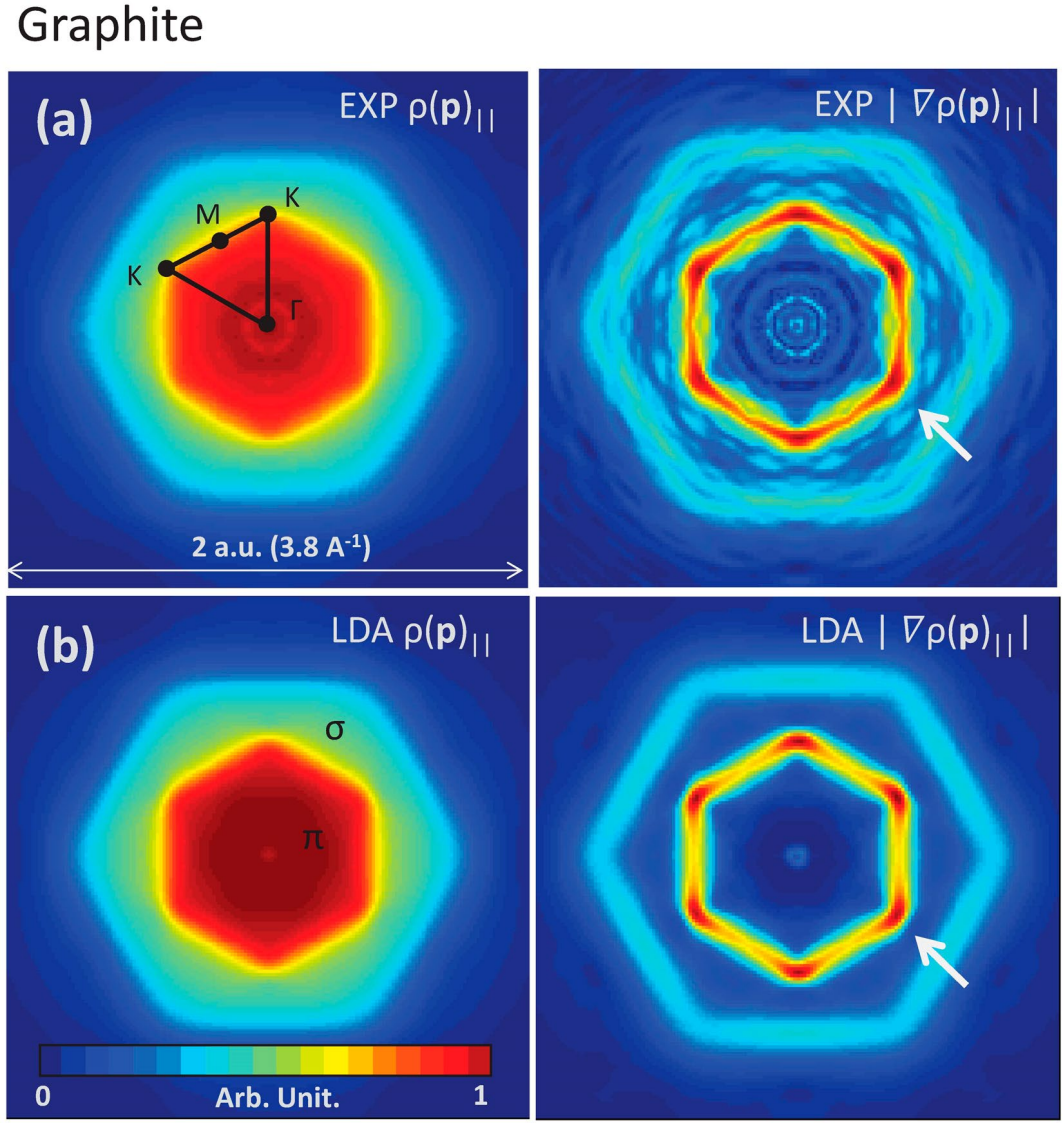
(**a**) Experimental EMDs in graphite and the derivatives and (**b**) those by band theory based on local density approximation. Arrows indicate Fermi-breaks at the Dirac points.

The questions we try to answer are now whether such an anisotropic broadening effect can be actually observed and how correctly the simple NFE model simulates it. To settle these questions, we need to perform an experiment, which is a rather challenging high-resolution experiment. There are several methods to measure the EMD. Angle-resolved photoemission spectroscopy (ARPES) widely used for band dispersion mapping could provide EMD if the intensity is integrated along the energy axis^12,13,24^. However, the transition matrix significantly varies depending on momentum and energy, and thus could be much inhomogeneous over **p**-space. Therefore this technique has an inherent problem for the present purpose. We therefore used Compton scattering. This technique provides EMD via tomographic reconstruction and has no such inhomogeneous effects^25,26^. Owing to the nature of the surface insensitivity, Compton scattering is recently attracting attention as a complementary method to ARPES^26–29^. However the scattering cross section is very small and almost no intensity would be observed on monolayer or bilayer samples. Therefore, we have focused on graphite (Kish graphite^30^). An LDA calculation was performed to ensure that graphite still has an anisotropic broadening effect [see, Figs 2(a) and 3(b)]. In addition, there is another critical problem. Compton scattering experiments are mostly performed with a momentum resolution of 0.2–0.3 Å^−1^ (~0.1–0.15 atomic units), but such a broad resolution would not clarify the anisotropic broadening. We have adopted a newly developed, high-resolution set-up (0.06 Å^−1^ resolution).

Figure 3a shows the EMD projected along the *c*-axis of graphite $\rho {({\bf{p}})}_{\parallel }$ measured by the experiment and the derivative $|\nabla \rho {({\bf{p}})}_{\parallel }|$. They are two-dimensionally reconstructed from five spectra or Compton profiles between the Γ-*K* and the Γ-*M* directions. The theoretical EMD is also reconstructed from five Compton profiles following the experimental procedure for the sake of an exact comparisons. They are based on standard band theory, adopting the local density approximation (LDA). As seen Fig. 3, those EMDs are basically a sum of two components of *π*(*p*_*z*_) electrons confined in the first zone and *σ*(*sp* ^2^) electrons in the second zone. Note that there are four *π* electrons while twelve *σ* electrons in the unit cell. Our present interest is in the former that forms the Dirac cones. The divergent behaviors are clearly seen at *K* on the $|\nabla \rho ({\bf{p}})|$ intensity map, indicating the momentum broadening as a function of the energy gap. This is exactly what we saw on the NFE model in graphene and the evidence of the anisotropic EMD broadening near the Dirac cones. Since Compton scattering detects the momentum density with a uniform sensitivity over a wide momentum range, other effects like an inhomogeneous cross-section effect would not produce such features.

## Discussion

We have demonstrated that EMD exhibits broadening as a function of the gap magnitude. Because of this nature, a unique topology of the EMD exists in graphene and graphite, as has actually been observed by the experiment. This behavior mimics the evolution of EMD across the metal-insulator transition. In insulators or semiconductors close to the transition point, the system has a small gap and thus gives an EMD representing the shape of the Brillouin zone. As the gap becomes larger, so too does the broadening, eventually leading to a rounded shape in the limit of *E*_*g*_ → ∞.

In graphene and graphite, the interference of wavefunctions via reciprocal lattice vectors causes the broadening effect. A similar argument may be extended to various cases, *e*.*g*., charge/spin density wave (CDW/SDW) materials or even superconductors. In fact, the earlier theoretical studies were mainly made for the latter^3–5^. The wavefunctions of the electrons in those materials interfere via nesting vectors due to the lattice distortion or via phonons (or other sources) mediating the Cooper paring and making an energy gap. Though the driving forces may be different, they commonly make an interference between two states and couple them. Since the broadening effect is effectively given as *E*_*g*_/v*, one can see Fermi-surface-associated features even in insulating or superconducting phases if the energy gap is sufficiently small. As the gap becomes larger, it should be more difficult to find them due to the strong broadening.

We also realize that the broadening effect would be significant when v* is small. Therefore the broadening may be more prominent in narrow-band materials, such as high-*T*_*c*_ cuprate superconductors or heavy Fermion systems. Compton scattering has already been applied to these systems but such a broadening effect has not been reported due to insufficient resolutions^27–29^. If the resolution is further improved, the broadening would be prominently detected across a metal-insulator transition.

The broadening effect should be commonly observed with other techniques based on momentum space, *e*.*g*., positron annihilation spectroscopy, probing two-particle (positron and electron) momentum densities. Indeed, there are earlier studies associated with this technique, discussing the momentum broadening across a CDW/SDW transition, superconducting transition, and so on^31–35^. In particular, studies for the electron localization in quantum dots are closely related to the present studies^32,33^. The observed momentum broadening has been attributed to the wide gap based on the theory above, although it is not possible to assess the validity of the theory itself because of an insufficient momentum resolution, possible positron trapping, and/or unknown Fermi velocities. The broadening effect also may be seen by ARPES if the momentum distribution curve (MDC) is carefully examined^36^. However, the inhomogeneity of the transition matrix could be more significant as already mentioned, and thus it might be difficult to isolate them from other contributions.

## Methods

The nearly-free-electron model was used to study the EMD in graphene. The periodic potential as a perturbation for the itinerant electrons was constructed by placing an atomic Coulomb potential with a charge number *Z* and an appropriate muffin-tin radius at each carbon site. The electron wavefunctions were expanded in terms of 545 planewaves. The two lowest-energy bands obtained in this way agree well with the *π*-bands from the first-principles electronic structure calculations. Since our model is considerably simple, it enables us to describe the electronic state in a highly controllable way: an energy gap can be opened at the Dirac points by placing impurity atoms with a charge *Z* = 0.5 at the positions indicated in Fig. 1c. The gap magnitude can be controlled with ease by changing the impurity charge number. Details are described in Supplemental Information.

The Compton scattering experiment was performed at the Taiwan IXS beamline at SPring-8 (BL12XU) at room temperature. The bent Laue spectrometer was used^37^. The incident photon energy was fixed at 25.5 keV while the scattered photon energy was scanned between 22.0 keV and 25.5 keV, corresponding to −4.4 and 6.6 atomic units in momentum, respectively. The scattering angle was 150°. The total instrumental momentum resolution was ~0.06 Å^−1^ (~0.03 atomic units). A Compton analysis is generally made based on the so-called impulse approximation, in which valence/core-hole effects are ignored^38,39^. The approximation is not entirely justified if the x-ray energy is low, causing an additional broadening effect. This natural broadening effect has been evaluated to be approximately one-third of the instrumental resolution in the same way as in an earlier report^40^. The sample was a single crystal of graphite (Kish graphite), having a size of 4 mm in height, 2 mm in width, and 0.1 mm in thickness. The 2D-EMD was reconstructed from five Compton profiles measured between the Γ-*M* and Γ-*K* directions. Details on the experiment and the reconstruction procedure are described in Supplemental Information.

Theoretical Compton profiles of graphite were calculated based on the first-principles band theory within LDA. The full-potential linearized augmented-planewave (FLAPW) method was adopted. The BANDS code developed in BL08W, SPring-8 was used. For a precise comparison with experiment, we first calculated five Compton profiles along the same axes on which the experiments were performed. The 2D-EMD was then reconstructed from them by exactly the same procedure as in the experiment.

## Electronic supplementary material

Supplementary info
