# Publisher Correction: Electron momentum densities near Dirac cones: Anisotropic Umklapp scattering and momentum broadening

**DOI:** 10.1038/s41598-017-15017-0

**Published:** 2017-11-10

**Authors:** N. Hiraoka, T. Nomura

**Affiliations:** 1 National Synchrotron Radiation Research Center (NSRRC), 101 Hsin-Ann Road, Hsinchu, 30076 Taiwan; 2 National Institutes for Quantum and Radiological Science and Technology (QST), SPring-8, 1-1-1 Kouto, Sayo, Hyogo 679-5148 Japan

*Scientific Reports* **7**:565; doi:10.1038/s41598-017-00628-4; Article published online 03 April 2017

The original Supplementary Information file published with this Article was incorrect. This version included errors in the titles of references 8 and 9 and a number of typographical errors. The correct Supplementary Information file now accompanies the Article.

