# Supplementary material for: Electron momentum densities near Dirac cones: Anisotropic Umklapp scattering and momentum broadening
Source: Sci Rep. 2017 Apr 3;7:565. doi: 10.1038/s41598-017-00628-4 (PMC5428786; doi:10.1038/s41598-017-00628-4)
Supplement: Supplementary file 1 — Supplementary info [file 41598_2017_628_MOESM1_ESM.pdf]

Supplementary Information on ‘Electron momentum densities near Dirac cones : Anisotropic Umklapp scattering and momentum broadening’

N. Hiraoka[1*] and T. Nomura[2]

[1]National Synchrotron Radiation Research Center (NSRRC), 101 Hsin-Ann Road, Hsinchu 30076, Taiwan
[2]National Institutes for Quantum and Radiological Science and Technology (QST), SPring-8, 1-1-1 Kouto, Sayo, Hyogo 679-5148, Japan
[*]hiraoka@spring8.or.jp

ABSTRACT

Supplementary Information is given for the report on ‘Electron momentum densities near Dirac cones : Anisotropic Umklapp scattering and momentum broadening’. The details for computations on the nearly free electron model, experiments of Compton scattering, and calculations upon band theory are described.

Nearly free electron model

Electrons travel in solids under the influence from a crystalline potential. This potential is described generally in the following form

$$U(\mathbf{r}) = \sum_{\mathbf{R}:\text{atoms}} U_{\text{atom}}(\mathbf{r}-\mathbf{R}), \tag{1}$$

where $\mathbf{R}$ runs over the positions of atoms forming the crystal. In the present study, we restrict ourselves to the cases of $U(\mathbf{r})$ possessing the same spatial periodicity as that of the crystal. This means that $U(\mathbf{r})$ can be expressed as: $U(\mathbf{r}) = \sum_{\mathbf{G}} U_{\mathbf{G}} \exp(i\mathbf{G}\cdot\mathbf{r})$, where $\mathbf{G}$'s are the reciprocal lattice vectors. As far as the potential is weak, we may regard the potential $U(\mathbf{r})$ as a perturbation to free electrons. The energy and wave functions of the unperturbed free electrons are parabolic and plane-wave functions respectively:

$$E^{(0)}_{\mathbf{k}} = \frac{\hbar^2\mathbf{k}^2}{2m}, \tag{2}$$

and

$$\psi^{(0)}_{\mathbf{k}}(\mathbf{r}) = \frac{1}{\sqrt{V}}\exp(i\mathbf{k}\cdot\mathbf{r}), \tag{3}$$

where m is the mass of the free electron, $\mathbf{k}$ is wave vector. To the lowest order in $U_{\mathbf{G}}$, the wave functions of perturbed states can be expanded using the unperturbed plane waves as

$$\psi_{\mathbf{k}}(\mathbf{r}) = \frac{1}{\sqrt{V}}\sum_{\mathbf{G}} C_{\mathbf{k}+\mathbf{G},\mathbf{k}}\,\psi^{(0)}_{\mathbf{k}+\mathbf{G}}(\mathbf{r}). \tag{4}$$

As found in standard textbooks on solid state physics[1,2], the coefficients $C_{\mathbf{k}+\mathbf{G},\mathbf{k}}$'s and perturbed electron energy $E_{\mathbf{k}}$ can be determined by the secular equation:

$$\begin{bmatrix} \ddots & \vdots & & \vdots & \\ \cdots & E^{(0)}_{\mathbf{k}} - E_{\mathbf{k}} & \cdots & U_{-\mathbf{G}} & \cdots \\ & \vdots & & \vdots & \\ \cdots & U_{\mathbf{G}} & \cdots & E^{(0)}_{\mathbf{k}+\mathbf{G}} - E_{\mathbf{k}} & \cdots \\ & \vdots & & \vdots & \ddots \end{bmatrix} \begin{bmatrix} \vdots \\ C_{\mathbf{k},\mathbf{k}} \\ \vdots \\ C_{\mathbf{k}+\mathbf{G},\mathbf{k}} \\ \vdots \end{bmatrix} = 0, \tag{5}$$

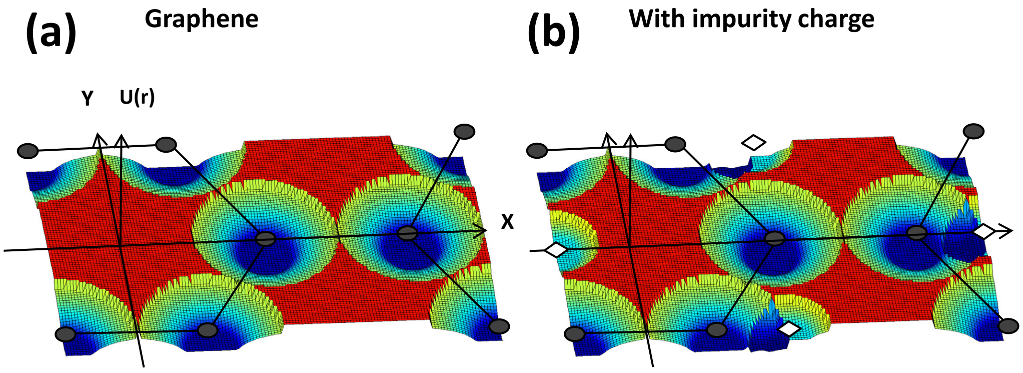

Figure S1. Crystalline potential $U(\mathbf{r})$ in graphene without impurities (left) and with impurities (right). Muffin-tin potentials are placed at the positions of carbon atoms (•) and impurities (◇).

Each solution of equation (5) is characterized by band index n as $E_{n\mathbf{k}}$ and $C_{\mathbf{k}+\mathbf{G},n\mathbf{k}}$.

We determine the wave function and electron energy by solving the above secular equation numerically at each wave vector $\mathbf{k}$. For numerical calculations, we take 545 $\mathbf{G}$'s, which are sufficient for precise determination of the lowest-energy bands. For the atomic Coulomb potential $U_{\text{atom}}(\mathbf{r})$, we assume a muffin-tin shape with cutoffs:

$$U_{\text{atom}}(\mathbf{r}) = \begin{cases} -Ze^2/r_{\text{min}} & (|\mathbf{r}| \leq r_{\text{min}}), \\ -Ze^2/|\mathbf{r}| & (r_{\text{min}} < |\mathbf{r}| < r_{\text{max}}), \\ 0 & (r_{\text{max}} \leq |\mathbf{r}|). \end{cases}$$

We control the strength of the potential by the charge number Z at each atom site. For carbon sites forming the honeycomb network, we take $r_{\text{min}} = 0.25$ (Å) and $r_{\text{max}} = 0.7$ (Å) (see Fig.S1). The two lowest-energy bands obtained by this way agree well with the π-bands by the first-principles electronic structure calculation. We can open the energy gap at the K points by introducing impurity atoms and breaking the crystalline inversion symmetry, as demonstrated in the text. For impurity sites, we take $r_{\text{min}} = 0.2$ (Å) and $r_{\text{max}} = 0.4$ (Å).

The electron momentum density (EMD) is defined by

$$\rho(\mathbf{p}) = \sum_{\mathbf{k}} \theta[E_F - E_{\mathbf{k}}] \left| \int d\mathbf{r} \exp[-i\mathbf{p}\cdot\mathbf{r}] \psi_{\mathbf{k}}(\mathbf{r}) \right|^2, \tag{6}$$

where E_F is the Fermi energy[3]. The Heaviside step function θ has been inserted for the sake of explicitness. To calculate $\rho(\mathbf{p})$ in equation (6), the reduced-zone scheme is more convenient. By substituting equation (4) into equation (6) and replacing the summation in $\mathbf{k}$ with that in band index n and reduced momentum $\bar{\mathbf{k}}$, we have finally

$$\rho(\mathbf{p}) = \sum_{n} \theta[E_F - E_{n\bar{\mathbf{p}}}] |C_{\mathbf{p},n\bar{\mathbf{p}}}|^2, \tag{7}$$

where $\bar{\mathbf{p}}$ is the reduced momentum for $\mathbf{p}$.

Dependence on impurity positions

A break of the symmetry due to impurity potentials easily makes a nearly isotropic energy gap and an isotropic momentum broadening. Although this is an artificial procedure, it is interesting to see the dependence on the impurity positions. First, we start the discussion with the case of no impurity [Fig.S2(a)]. This is same to the one in the main article. The anisotropic momentum broadening is exhibited: A sharp drop of the momentum density at the Dirac points or the K/K' points while a smooth fall at others are clearly seen. Shifting the impurities along the x-axis to a small extent, the system transits to an insulator [Fig.S2(b)]. The characteristic feature around the Dirac points disappears while a rectangular feature is added more or less. If we shift the impurities further, they overlap with some of the regular carbon atoms, resembling the potentials in boron nitride [Fig.S2(c)]. Then, drastic changes are seen: Flat bands having a gap as wide as 20 eV are made, mimicking a situation of an infinite gap. The momentum density are very board, only leaving a round shape. The hexagonal shape supposedly existing due to the crystal symmetry is hardly discernible. The next one [Fig.S2(d)] is identical with the one in the main article. An

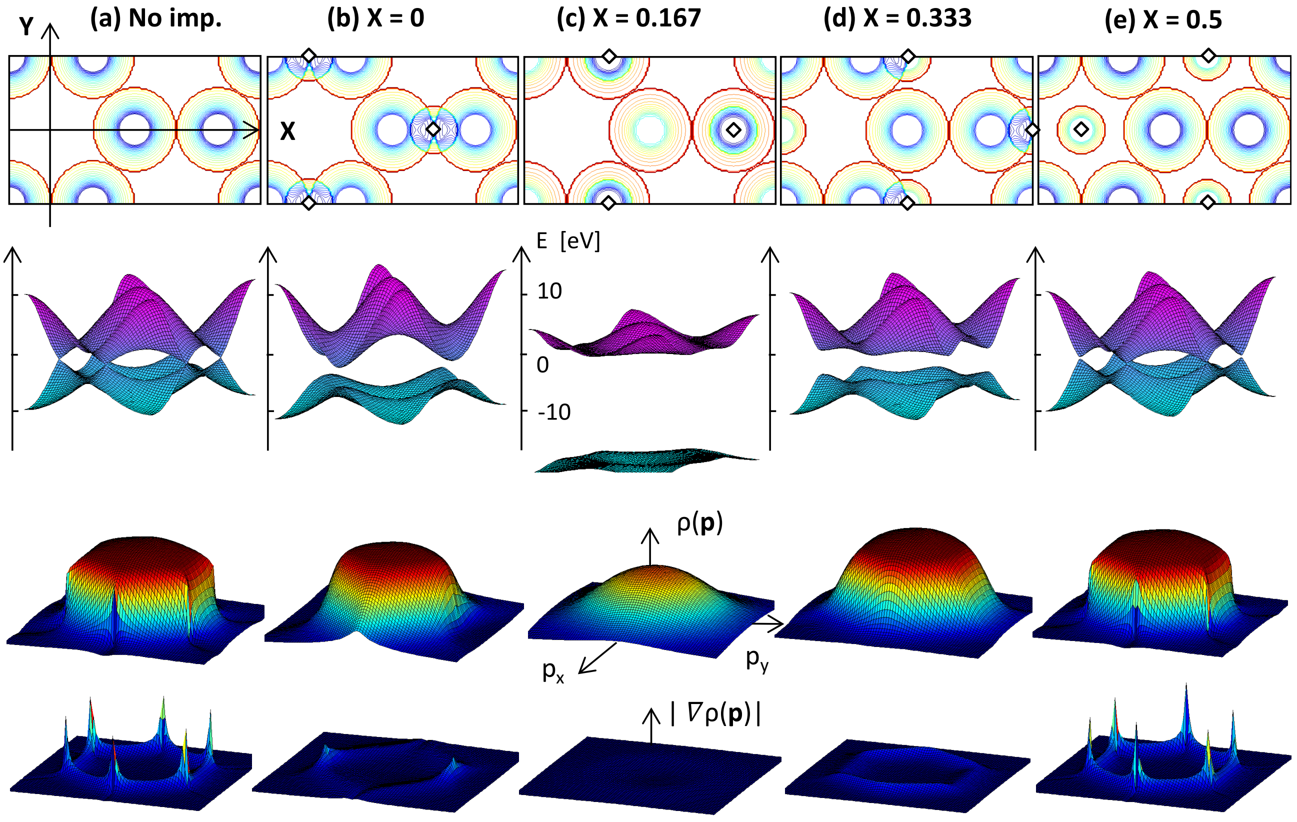

Figure S2. NFE band structures and momentum broadening as a fuction of impurity positions (◇). Top: Contour maps for the potentials, Middle: NFE occupied and unoccupied bands, Bottom: momentum density $\rho(\mathbf{p})$ and the derivatives $|\nabla\rho(\mathbf{p})|$. The scales are common for all the cases.

almost isotropic gap and the momentum broadening are recognized. Another interesting case appears when the impurities center at the hexagons with the carbon atoms [Fig.S2(e)]. Since the symmetry is the same to that in graphene, very similar band structures are obtained despite additional potentials. At the same time, the characteristic feature at K/K' points in the momentum density reappears, rendering the strikingly anisotropic broadening.

Experimental/Theoretical Compton profiles

An energy spectrum of Compton scattered x-rays has a broad feature, representing the distribution of electron momentum densities (EMDs) $\rho(\mathbf{p})$ in the scatterer[3,4]. Within the impulse approximation[5,6], the observable quantity is the double integral of the EMDs projected onto the scattering vector, *i.e.,* Compton profile $J(p_z)$;

$$J(p_z) = \int\int \rho(\mathbf{p})\, dp_x dp_y. \tag{8}$$

Here p_z is taken as an electron momentum component parallel to the scattering vector $\mathbf{q}$, associated with the scattered photon energy ε with the following equation :

$$\varepsilon_o = \varepsilon + \frac{(\hbar q)^2}{2m} + p_z \frac{|\hbar \mathbf{q}|}{m}. \tag{9}$$

Here, ε_o is the incident photon energy, $\hbar$ the Plank constant, and m the electron rest mass. The original $\rho(\mathbf{p})$ is restored by a tomographic reconstruction from dozens of Compton profiles measured on different axes. What we attempted in this study is

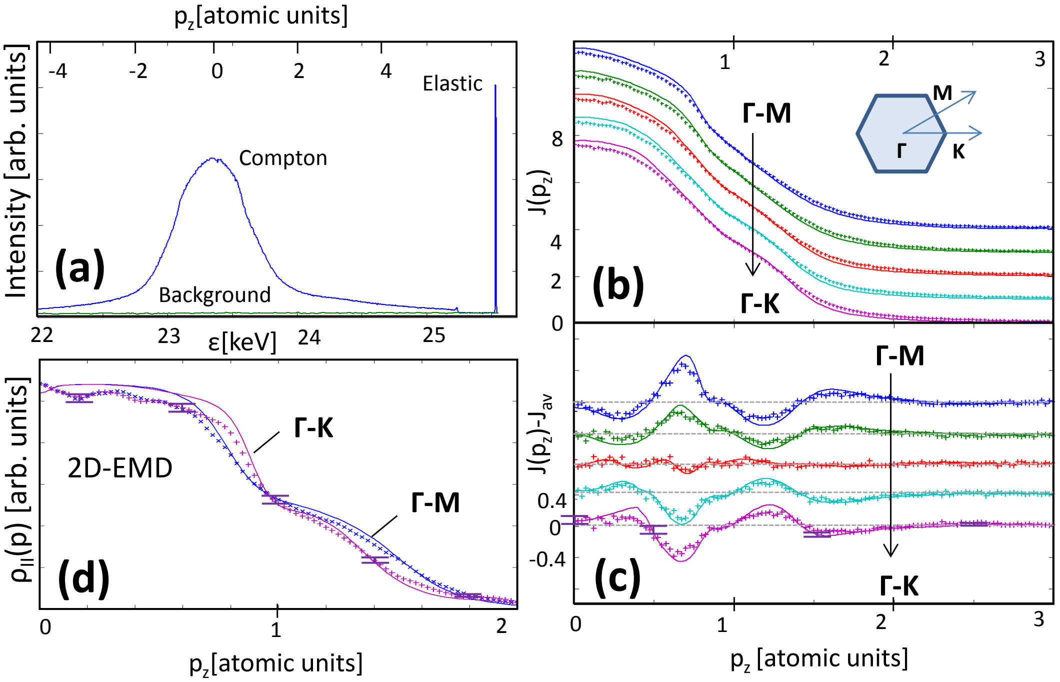

Figure S3. **(a)** Example of obtained spectra, **(b)** Directional Compton profiles $J(p_z)$ and **(c)** their anisotropies $J(p_z)$-J_{av} by the experiment (+) along with band theory (−), where J_{av} is the average of the five Compton profiles. The profiles are shifted along the vertical axes for easier comparisons. The statistical errors in **(b)** are smaller than the marker size. **(d)** 2D-reconstructed experimental EMD's along Γ-M (×) and Γ-K (+), along with LDA theory (−).

the two-dimensional reconstruction. The obtained quantity is a projected on-plane EMD;

$$\rho_{||}(p_x, p_y) = \int \rho(\mathbf{p})\, dp_z. \tag{10}$$

Note that the scattering vectors are now on the p_x-p_y plane and thus they are orthogonal to p_z, unlike in the equation (9). The advantage of this approach is that only several Compton profiles are necessary for the reconstruction and the computing efforts and artifacts are much less, compared to the full (3D) reconstruction. Although there remains one integral but this approach nicely works for 2D systems just like graphite. The details of the procedure are described in the earlier reports.[7–9]

The experiment was performed at the Taiwan IXS beamline at SPring-8 (BL12XU). With a Si(111) double crystal monochromator and a Si(660) bent Laue spectrometer[10], we had a 5 eV resolution at 25.54 keV (ε_o), which corresponds to 0.030Å^{-1} or 0.016 atomic units (a.u.) momentum resolution. The scattering angle was 150°. A vertical slit was installed between the sample and the analyzer, (10-mm wide, 750mm away from the sample). This finite width make the angular uncertainty of 0.013 rad, leading to an additional broadening of 0.049 Å^{-1} (0.026 a.u.). The total resolution in instrumentation was 0.057 Å^{-1} (0.030 a.u.). The impulse approximation, in which any core/valence hole effect is neglected to obtain equation (8), is not entirely fulfilled if the x-ray energy ε_o is low or the momentum transfer $\mathbf{q}$ is small. This final state effect also leads to a broadening effect.[11] We examined this effect on isotropic systems, Na and Li, and found out a 3-4 eV broadening, which corresponds to ~0.02 Å(0.01 a.u.).

The sample was a single crystal of graphite (Kish graphite), having a size of 4-mm in height, 2-mm in width, and 0.1-mm in thickness. We measured five Compton profiles between Γ-M and Γ-K directions (see Fig.S3). Approximately 50 000 counts were accumulated at the peak of each profile for an exposure time of 12 hours. They were corrected for the scattering cross section, the efficiency of the spectrometer, the self and air absorption, and the multiple scattering events. They were then normalized with an electron number and folded at p_z=0 in order to make the final forms of the Compton profiles. Finally the core (1s) contribution were subtracted before the reconstruction. Figure S3 displays the experimental Compton profiles along with theory.

Theoretical Compton profiles of graphite were calculated based on a first-principles band theory. The BANDS code available in BL08W at SPring-8 was used. The crystal symmetry was D_{6h}^4 (No.194). The lattice parameters were 2.464 Å and 6.711 Å for the a- and c-axes, respectively. The muffin-tin (MT) radius was 0.70 Å (1.32 a.u.). The formula by Vosko, Wilk,

and Nusair were used for local density approximation (LDA)[12]. The full-potential linearized augmented-plane-wave (FLAPW) method was used for the description of the electron wavefunctions. The angular momentum expansion is truncated at $\ell_{max} = 7$, for the wavefunctions, and at 6 for the charge density and the potential. We sampled 57 k-points in the irreducible Brillouin zone. The obtained band structure was confirmed to be identical with that in an earlier report.[13] For a precise comparison with experiment, we first calculated five Compton profiles along the axes on which the experiments were done. The 2D-EMDs were then reconstructed from them by the exactly same procedure as the experiment.

References

1. Ashcroft, N. W. & Mermin, N. D. *Solid State Physics, Chapter 9* (Thomson Learning, Inc., 1976).
2. Ziman, J. M. *Principles of The Theory of Solids: 2nd edition, Chapter 3* (Cambridge University Press, 1972).
3. Schülke, W. *Electron Dynamics by Inelastic X-ray Scattering: Chapter 4* (Oxford University Press, 2007).
4. Cooper, M. J., Mijnarends, P. E., Shiotani, N., Sakai, N. & Bansil, A. *X-ray Compton Scattering: Chapter 1* (Oxford University Press, 2004).
5. Eisenberger, P. & Platzman, P. M. Compton scattering of x-rays from bound electrons. *Phys. Rev. A* **2**, 415–423 (1970).
6. Kaplan, I. G., Barbiellini, B. & Bansil, A. Compton scattering beyond the impulse approximation. *Phys. Rev. B* **68**, 235104 (2003).
7. Tanaka, Y. *et al.* Reconstructed three-dimensional electron momentum density in lithium: A Compton scattering study. *Phys. Rev. B* **63**, 045120 (2001).
8. Hiraoka, N., Buslaps, T., Honkimäki, V., Guyot, H. & Schlenker, C. Hidden one dimensionality in Fermi surfaces of η-Mo_4O_{11} observed by Compton scattering experiments. *Phys. Rev. B* **71**, 125417 (2005).
9. Hiraoka, N. *et al.* Momentum densities, Fermi surfaces, and their temperature dependences in Sr_2RuO_4 studied by Compton scattering. *Phys. Rev. B* **74**, 100501(R) (2006).
10. Hiraoka, N. *et al.* An x-ray Raman spectrometer for EXAFS studies on minerals: bent Laue spectrometer with 20 kev x-rays. *J. Synchrotron Rad.* **20**, 266–271 (2013).
11. Soininen, J. A., Hämäläinen, K. & Manninen, S. Final-state electron-electron interaction in Compton scattering. *Phys. Rev. B* **64**, 125116 (2001).
12. Vosko, S. H., Wilk, L. & Nusair, M. Accurate spin-dependent electron liquid correlation energies for local spin density calculations: a critical analysis. *Can. J. Phys.* **58**, 1200–1211 (1980).
13. Marinopoulos, A. G., Reining, L., Rubio, A. & Olevano, V. *Ab initio* study of the optical absorption and wave-vector-dependent dielectric response of graphite. *Phys. Rev. B* **69**, 245419 (2004).
